# Supplementary material for: Heavy metal exposure and its impact on inflammatory ratios in minors: The mediating role of BMI
Source: PLoS One. 2025 Dec 29;20(12):e0339470. doi: 10.1371/journal.pone.0339470 (PMC12747377; doi:10.1371/journal.pone.0339470)
Supplement: Attachment — Submitted filename: PlosOne comments.pdf [file pone.0339470.s003.pdf]

his manuscript investigates the associations between heavy metal exposure (Pb, Cd, Hg) and inflammatory ratios in minors using NHANES 2007–2018 data, and evaluates BMI as a potential mediator. The topic is relevant to environmental health and pediatric epidemiology, and the authors present a large dataset with several analytical approaches.

Concern: The use of adult BMI cutoffs in a pediatric population, which affects all interpretations involving BMI.

Grammer mistakes in Abstract background: **Healthy of minors to health of minors**
