# Supplementary material for: Heavy metal exposure and its impact on inflammatory ratios in minors: The mediating role of BMI
Source: PLoS One. 2025 Dec 29;20(12):e0339470. doi: 10.1371/journal.pone.0339470 (PMC12747377; doi:10.1371/journal.pone.0339470)
Supplement: Attachment — Submitted filename: Comments.docx [file pone.0339470.s001.docx]

**Comments to the author:**

The manuscript **“Heavy Metal Exposure and Its Impact on Inflammatory Ratios in Minors: The Mediating Role of BMI**” offers insight into the effect of heavy metals on the health of the minors which has been exclusively studied in adults till now, imparting novelty to the present work. But the manuscript needs certain modifications to enhance its suitability for publication in this journal, which are listed below:

1. Manuscript needs to be checked by native English speaker to negate any language related errors, in result section, prefer to keep the same verb tense which is fluctuating from past to simple present tense.

2. The abbreviations for neutrophil-to-lymphocyte ratio, lymphocyte-to-monocyte ratio, platelet-to-lymphocyte ratio, platelet-to-neutrophil ratio, and neutrophil-to-monocyte ratio have been mentioned in introduction section. Do not specify the same in section 2.3 (line no 102).

3. Include the significance of the inflammatory markers for better understanding of the relationship between heavy metals and inflammatory markers.
