# Supplementary material for: Heavy metal exposure and its impact on inflammatory ratios in minors: The mediating role of BMI
Source: PLoS One. 2025 Dec 29;20(12):e0339470. doi: 10.1371/journal.pone.0339470 (PMC12747377; doi:10.1371/journal.pone.0339470)
Supplement: Attachment — Submitted filename: Response to Reviewers.docx [file pone.0339470.s004.docx]

**Manuscript ID:** PONE-D-25-21195

**Manuscript Title:** Heavy Metal Exposure and Its Impact on Inflammatory Ratios in Minors: The Mediating Role of BMI

**Authors:** Xinpeng Li, Lu Han*

The authors appreciate the professional comments from the editors and reviewers. According to the comments, we have made extensive modifications to our manuscript to make our results convincing. Our point-by-point responses were presented as follows:

**Response to Reviewer 1:**

Reviewer #1: The manuscript “Heavy Metal Exposure and Its Impact on Inflammatory Ratios in Minors: The Mediating Role of BMI” offers insight into the effect of heavy metals on the health of the minors which has been exclusively studied in adults till now, imparting novelty to the present work. But the manuscript needs certain modifications to enhance its suitability for publication in this journal, which are listed below:

1. Manuscript needs to be checked by native English speaker to negate any language related errors, in result section, prefer to keep the same verb tense which is fluctuating from past to simple present tense.

Response 1: We sincerely appreciate the reviewer’s valuable suggestion. In response, we have carefully refined the language throughout the manuscript to improve clarity and precision without altering the original meaning. Furthermore, the verbs in the **Methods and Results** sections have been consistently revised to the past tense where appropriate. All these revisions are clearly highlighted in the tracked changes of the revised manuscript.

2. The abbreviations for neutrophil-to-lymphocyte ratio, lymphocyte-to-monocyte ratio, platelet-to-lymphocyte ratio, platelet-to-neutrophil ratio, and neutrophil-to-monocyte ratio have been mentioned in introduction section. Do not specify the same in section 2.3 (line no 102).

Response 2: We sincerely thank the reviewer for the insightful comment. The abbreviations PC, NC, LC, and MC in **Section 2.3 (line 102)** refer to platelet count, neutrophil count, lymphocyte count, and monocyte count, respectively. These abbreviations differ from those defined earlier in the **Introduction** (i.e., NLR, LMR, PLR, PNR, and NMR), which represent ratio-based indicators. As PC, NC, LC, and MC appear for the first time in this section, we have retained their definitions here for clarity.

3. Include the significance of the inflammatory markers for better understanding of the relationship between heavy metals and inflammatory markers.

Response 3: We have added a description in **lines 72–78** of the **Introduction** section, emphasizing the close association between heavy metal exposure, disruption of immune homeostasis, and the promotion of systemic inflammation. This revision helps readers better understand the relationship between heavy metals and inflammatory responses.

**Response to Reviewer 2:**

Reviewer #2: This study explores the relationship between heavy metal exposure and inflammatory ratios in minors, with BMI as a potential mediator, using NHANES 2007–2018 data. The large sample size and age-group stratification strengthen the findings. The use of linear regression and mediation analysis is appropriate and reveals consistent patterns: blood Pb, Cd, and Hg are significantly associated with inflammatory ratios, and BMI mediates 20–40% of these effects. The results are clearly presented and the conclusions are supported by the data. I recommend that the authors expand the methodology and figure legends.

Response: We have added a detailed description of the mediation effect in **Section 2.6 “Mediation analysis”** of the **Methods, lines 135-147**. In addition, we have revised and enriched the contents to provide clearer explanations of variables, models, and significance indicators in **Figure 4 (Section 3.5, lines 246–256)**. The legend for Figure 4 has also been made more detailed to improve clarity, **lines 257-260**.
